# Left Frontoparietal Control Network Connectivity Moderates the Effect of Amyloid on Cognitive Decline in Preclinical Alzheimer’s Disease: The A4 Study

**DOI:** 10.14283/jpad.2024.140

**Published:** 2024-07-24

**Authors:** R. Boyle, H. M. Klinger, Z. Shirzadi, G. T. Coughlan, M. Seto, M. J. Properzi, D. L. Townsend, Z. Yuan, C. Scanlon, R. J. Jutten, K. V. Papp, R. E. Amariglio, D. M. Rentz, J. P. Chhatwal, M. C. Donohue, R. A. Sperling, A. P. Schultz, Rachel F. Buckley, A4 Study Team

**Affiliations:** 1 Department of Neurology, Harvard Aging Brain Study, Massachusetts General Hospital, Harvard Medical School, Boston, MA 02114 USA; 2 Department of Neurology, Center for Alzheimer Research and Treatment, Brigham and Women’s Hospital, Harvard Medical School, Boston, MA 02115 USA; 3 Alzheimer’s Therapeutic Research Institute, Keck School of Medicine, University of Southern California, San Diego, CA 90033 USA; 4 Athinoula A. Martinos Center for Biomedical Imaging, Department of Radiology, Massachusetts General Hospital, Charlestown, MA 02129 USA; 5 Melbourne School of Psychological Sciences, University of Melbourne, Melbourne, VIC 3010 Australia; 6 https://www.actcinfo.org/

**Keywords:** Cognitive resilience, functional connectivity, functional magnetic resonance imaging positron emission tomography (PET), β-amyloid

## Abstract

**Background:**

Stronger resting-state functional connectivity of the default mode and frontoparietal control networks has been associated with cognitive resilience to Alzheimer’s disease related pathology and neurodegeneration in smaller cohort studies.

**Objectives:**

We investigated whether these networks are associated with longitudinal CR to AD biomarkers of beta-amyloid (Aβ).

**Design:**

Longitudinal mixed.

**Setting:**

The Anti-Amyloid Treatment in Asymptomatic Alzheimer’s Disease (A4) study and its natural history observation arm, the Longitudinal Evaluation of Amyloid Risk and Neurodegeneration (LEARN) study.

**Participants:**

A sample of 1,021 cognitively unimpaired older adults (mean age = 71.2 years [SD = 4.7 years], 61% women, 42% APOEε4 carriers, 52% Aβ positive).

**Measurements:**

Global cognitive performance (Preclinical Alzheimer’s Cognitive Composite) was assessed over an average 5.4 year follow-up period (SD = 2 years). Cortical Aβ and functional connectivity (left and right frontoparietal control and default mode networks) were estimated from fMRI and PET, respectively, at baseline. Covariates included baseline age, APOEε4 carrier status, years of education, adjusted gray matter volume, head motion, study group, cumulative treatment exposure, and cognitive test version.

**Results:**

Mixed effects models revealed that functional connectivity of the left frontoparietal control network moderated the negative effect of Aβ on cognitive change (p =.025) such that stronger connectivity was associated with reduced Aβ-related cognitive decline.

**Conclusions:**

Our results demonstrate a potential protective effect of functional connectivity in preclinical AD, such that stronger connectivity in this network is associated with slower Aβ-related cognitive decline.

**Electronic Supplementary Material:**

Supplementary material is available in the online version of this article at 10.14283/jpad.2024.140.

## Introduction

Cognitive resilience (CR) refers to a property of the brain that enables better-than-expected cognitive performance given age-related brain changes, injury or disease (1). In preclinical AD, higher CR is associated with delayed clinical progression (2–4). The idea that differences in integrity and connectivity of functional networks may account for heterogeneity in the clinical expression of pathology (5) has motivated the use of resting-state fMRI (rs-fMRI) to identify neural correlates of CR (5–7).

Our team previously reported findings from the Harvard Aging Brain Study that stronger connectivity of the frontoparietal control network (FPCN) and default mode network (DMN) moderated the effect of beta-amyloid (Aβ) on cognitive decline over a median 3 year follow-up period (8). Individuals with FPCN or DMN connectivity showed reduced cognitive decline compared to individuals with weaker connectivity, at similar levels of Aβ. Similar protective effects have been detected at the cross-section whereby stronger FPCN connectivity was associated with better cognitive performance at similar levels of AD pathology (9–11). These findings suggested that the FPCN connectivity-CR association may be specific to the left FPCN (9).

It is now clear that large sample sizes are needed to identify robust associations between connectivity and cognition as smaller sample sizes can lead to inflated effect sizes and non-replicable results (12). This poses a challenge for studying CR to AD, as few studies have large samples with both rs-fMRI and PET imaging available. The Anti-Amyloid Treatment in Asymptomatic Alzheimer’s Disease (A4) study is the first secondary prevention trial of an anti-Aβ therapy, solanezumab, in clinically normal older adults at high risk for cognitive decline due to elevated Aβ-PET burden (13). The placebo arm of this study, and the adjacent natural history observation arm, (the Longitudinal Evaluation of Amyloid Risk and Neurodegeneration (LEARN) study), includes rs-fMRI, PET and repeated cognitive assessments in over 1,000 individuals and provides a unique opportunity to assess the moderating effect of FPCN and DMN connectivity on the association between Aβ and cognitive decline for up to 9 years in some individuals. We focused on the placebo arm to avoid treatment bias that may confound or obscure our results. Based on prior evidence, we hypothesized that stronger connectivity, specifically in the left FCPN, would be associated with an attenuated effect of Aβ on cognitive decline.

## Methods

### Participants

Eligible participants for the A4 study were cognitively unimpaired adults aged between 65–85 years old with elevated Aβ levels on screening PET. Normal cognitive function was defined based on these criteria for adults with ≥ 13 years of education: Clinical Dementia Rating (CDR) score = 0, Mini-Mental State Examination (MMSE) score = 27–30, Wechsler Memory Scale Logical Memory IIa sub-test - Delayed Recall (Logical Memory) score between 8–15; and the following criteria for adults with ≤ 12 years of education: CDR score = 0, MMSE score = 25–30, Delayed Recall score = 6–13. Elevated Aβ levels were classified using a visual read and quantitative measurement (standardized uptake value ratio; SUVr ≥ 1.15 in a template based set of cortical regions) of 18^F^ florbetapir(FBP)-PET scans (14, 15). We restricted our sample to participants who were randomized into the placebo treatment arm as well as participants from the natural history observational arm, the LEARN study. From an initial 1,703 participants in the pre-randomization fMRI dataset, participants were excluded based on fMRI QA metrics (n = 40); if they only completed a single cognitive assessment (n = 86); if they had missing data for the following variables: scanner vendor (n = 7), gray matter volume (n = 6), APOE ε4 carrier status (n = 10), treatment dose (n = 3); and if they were assigned to the A4 treatment group (n = 530). In total, there were 12,391 cognitive assessments across the 1,021 participants retained in our final analyses.

### Resting-state fMRI

Acquisition parameters differed across A4/LEARN sites as different vendors were used (GE Medical Systems, Phillips Medical Systems, Siemens Medical Systems; flip angle = 80–90°; slice thickness = 3.3–4 mm; TR = 2920–4030 ms; TE = 30 ms). The most common sequence was a Siemens scan (flip angle = 80°; slice thickness = 3.3 mm; TR = 3000 ms; TE = 30 ms). Resting-state fMRI data from the pre-randomization baseline dataset (i.e. data acquired at screening/baseline, prior to randomization into treatment or placebo arms) were preprocessed using SPM12 (fil.ion.ucl.ac.uk/spm/) following a previously described in-house pipeline (16), that included realignment, direct normalization, spatial smoothing with a 6-mm FWHM Gaussian kernel, and quality assessment on pre-defined thresholds (see Supplementary Methods 1.1).

**Figure 1:**
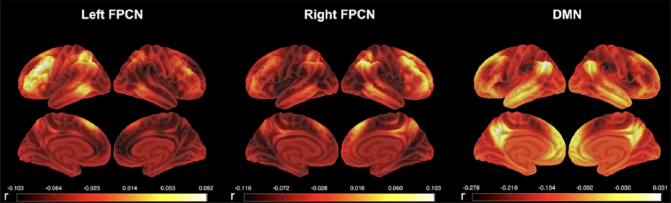
Average functional connectivity in the three networks of interest in the A4 and LEARN cohorts: left frontoparietal control network (Left FPCN), right frontoparietal control network (Right FPCN), and default mode network (DMN)

Functional connectivity estimates for the left FPCN, right FPCN, and DMN were obtained using the Template Based Rotation (TBR) method (16). TBR maps variance from individual volumes to out-of-sample spatial templates and we examined network templates corresponding to the FPCN and DMN. The FPCN was parcellated into two lateralized templates (left and right FPCN) and, as such, both templates were analyzed separately. Whole network measurements were made using a spatial correlation approach (17). Briefly, this was accomplished by a voxel-to-voxel correlation between the template maps and the individual subject maps produced by TBR. Key left and right FPCN nodes included the lateralized superior parietal lobule, inferior temporal and dorsolateral prefrontal cortices as well as the presupplementary motor area (see Fig. 1). Key DMN nodes included the posterior cingulate, medial prefrontal, bilateral middle temporal, bilateral parahippocampal cortices, the bilateral angular gyrus and the anti-correlated salience network nodes, namely the bilateral insula, bilateral middle prefrontal cortex, bilateral supramarginal gyrus, anterior cingulate cortex (see Fig. 1).

### Aβ-PET

18^F^-Florbetapir FBP-PET scans were acquired 50–70 minutes post-injection of the contrast agent. As outlined in Insel et al (18), FBP-PET images were realigned, averaged, and normalized to template space. We used a cortical neocortical composite SUVr referenced to the whole cerebellum (19). We used FBP-PET data from the pre-randomization baseline dataset.

### Cognitive Performance

Cognitive performance was assessed using the Preclinical Alzheimer Cognitive Composite (20) (PACC), which is sensitive to Aβ-related cognitive decline and is a composite of z-scores from four measures (20, 21): Free and Cued Selective Reminding Test - sum of Free and Total Cued Recall score (FCSRT); Logical Memory; Wechsler Adult Intelligence Scale-Revised - Digit Symbol Substitution Test score (DSST); MMSE. Alternate versions of the component measures were used across visits to minimize practice effects (13, 22) and a covariate representing PACC version was included in our analyses.

### Open-Label Extension (OLE) period

Participants in the A4 placebo arm were able to proceed to Solanezumab treatment during the OLE period. To account for any residual treatment effects during the OLE period, we calculated cumulative treatment dose as the cumulative sum of treatment dose received at each time point.

### Statistical Analyses

We first assessed independent associations of Aβ and functional connectivity in each network with PACC performance at the baseline in linear regressions adjusting for covariates including age, APOE ε4 status (carrier vs non-carrier), years of education, and head motion. To assess whether functional connectivity of the left FPCN, right FPCN, and DMN moderated the effect of Aβ on cognitive decline, we adapted the primary endpoint analysis from the A4 trial, which used a spline basis expansion of time (natural cubic splines with 2 degrees of freedom) to model change in PACC. We investigated separate mixed effects models with longitudinal PACC as the outcome variable including the two-way Aβ-by-time interaction (Aβ-by-time), the two-way functional connectivity-by-time interaction (FC-by-time) and finally, the three-way functional connectivity-by-time-by-Aβ interaction effect (FC-by-time-by-Aβ). We assessed statistical significance using likelihood ratio tests comparing each two- or three-way interaction effect model against reduced models. Covariates in all models included age, ε4 status (carrier vs non-carrier), years of education, adjusted GM volume, head motion, study group (A4 vs LEARN), cumulative treatment dose, and PACC version. All models included random intercepts (participant nested within MRI scanner [n = 70 unique scanners]) and random slopes terms. We conducted additional sensitivity analyses to fully interrogate the three-way interaction effects (see Supplementary Methods 1.2 for further detail on statistical analyses).

Statistical analyses were conducted in R using the lm function from the stats package and the lme function from the nlme package and statistical models were visualized using the plot_model function from the sjPlot package.

### Data Availability

Anonymized pre-randomization baseline data are publicly available by request through LONI (ida.loni.usc.edu/login.jsp?project=A4).

## Results

### Cohort characteristics

A4 placebo arm participants and LEARN participants were an average of 71.2 (4.7) years old with over 16 years of education (see Table 1) and completed 12 PACC assessments on average. Participants were assessed over an average duration of 5.4 years follow-up (see Supplementary Table 1 for distribution of follow-up periods). The length of follow-up period was not associated with baseline connectivity values, baseline PACC, or PACC at final visit. Better model fit of PACC with a spline basis expansion of time (AIC = 48,684.4) vs PACC with a linear time trend confirmed that a spline model was appropriate to capture a non-linear group level trend for PACC change (see Supplementary Fig. 1).

**Table 1:** Descriptive statistics

**Characteristic**	**A4 Placebo N = 532**	**LEARN N = 489**	**Overall N = 1,021**^**1**^	**p-value**
Age (Years)	71.8 (4.9) [65, 85.5]	70.6 (4.4) [65, 85.6]	71.2 (4.7) [65, 85.6]	<.001
Education (Years)	16.6 (2.9) [8, 30]	16.7 (2.6) [8, 30]	16.6 (2.8) [8, 30]	.3
Female	324 (61%)	303 (62%)	627 (61%)	.7
APOE ε4 Carrier	314 (59%)	113 (23%)	427 (42%)	<.001
PACC	−1.2 (2.4) [−11.2, 5]	−0.6 (2.2) [−8.1, 5.5]	−0.9 (2.2) [−11.2, 5.5]	<.001
PACC Timepoints	12.6 (4.5) [2, 21]	11.6 (4) [2, 18]	12.1 (4.3) [2, 21]	<.001
Weeks from PACC baseline	283.5 (113) [10, 468.1]	278.2 (106.8) [6.4, 434.9]	281 (110) [6.4, 468.1]	.3
Cortical Aβ (SUVR)	1.3 (0.2) [1, 2.1]	1 (0.1) [0.8, 1.2]	1.2 (0.2) [0.8, 2.1]	<.001
Adj. GM Volume (mm^3^)	578,939.5 (30,127.4) [500,052.4, 706,964.8]	580,862 (28,517.4) [477,907.5, 678,366.7]	579,860.3 (29,368.7) [477,907.5, 706,964.8]	.3
DMN FC	0.6 (0.1) [0.3, 0.7]	0.6 (0.1) [0.3, 0.7]	0.6 (0.1) [0.3, 0.7]	.7
Left FPCN FC	0.5 (0.1) [0.2, 0.6]	0.5 (0.1) [0.3, 0.6]	0.5 (0.1) [0.2, 0.6]	.026
Right FPCN FC	0.5 (0.1) [0.3, 0.7]	0.5 (0.1) [0.3, 0.6]	0.5 (0.1) [0.3, 0.7]	.015
Mean FWD (mm)	0.1 (0.1) [0.01, 0.4]	0.1 (0.1) [0.02, 0.43]	0.1 (0.1) [0.01, 0.4]	.2
Scanner Vendor				.029
Siemens	67%	75%	71%	
GE Medical Systems	24%	19%	22%	
Philips	9%	6%	7%	

1. Mean (SD) [Range]; n (%)

### Independent associations of Aβ and functional connectivity with baseline PACC performance and PACC change over time

Adjusting for age, APOE ε4 status, years of education, and head motion, baseline PACC performance was negatively associated with Aβ (p < .001, see Supplementary Table 2). Baseline PACC performance was not associated with connectivity of the left FPCN (p = .644), right FPCN (p = .775), or DMN (p = .752), independent of age, APOE ε4 status, years of education, head motion, and Aβ (see Supplementary Table 3).

**Figure 2:**
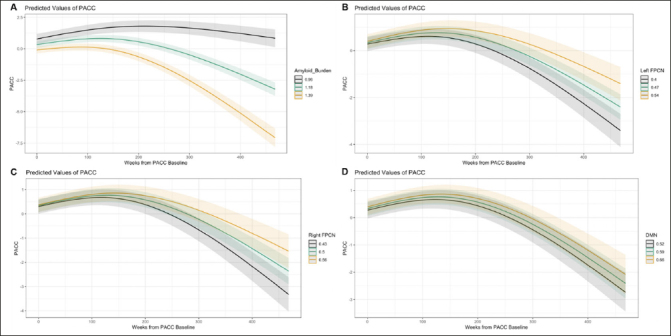
Marginal effects plots for the associations between Aβ or functional connectivity with PACC change (A: Aβ, B: Left FPCN, C: Right FPCN, D: DMN) Note: Marginal effects of the Aβ OR connectivity-by-time interaction are plotted here when cumulative dose is held constant at 0, continuous variables are equal to their mean values, and factor variables equal their reference values (ε4 status = non-carrier, group = A4 study; PACC version = Version A).

Aβ, left FPCN, and right FPCN had statistically significant curvilinear relationships with PACC change over time (see Supplementary Tables 4 and 5 for full mixed effects models and Supplementary Table 6 for likelihood ratio tests). Higher levels of Aβ were associated with faster cognitive decline (see Fig. 2A). By contrast, stronger connectivity of the left FPCN or right FPCN was associated with less cognitive decline (see Fig. 2B-D). The association between DMN connectivity and PACC change over time was not statistically significant.

### Left frontoparietal control network functional connectivity moderates the effect of Aβ on PACC change over time

**Figure 3:**
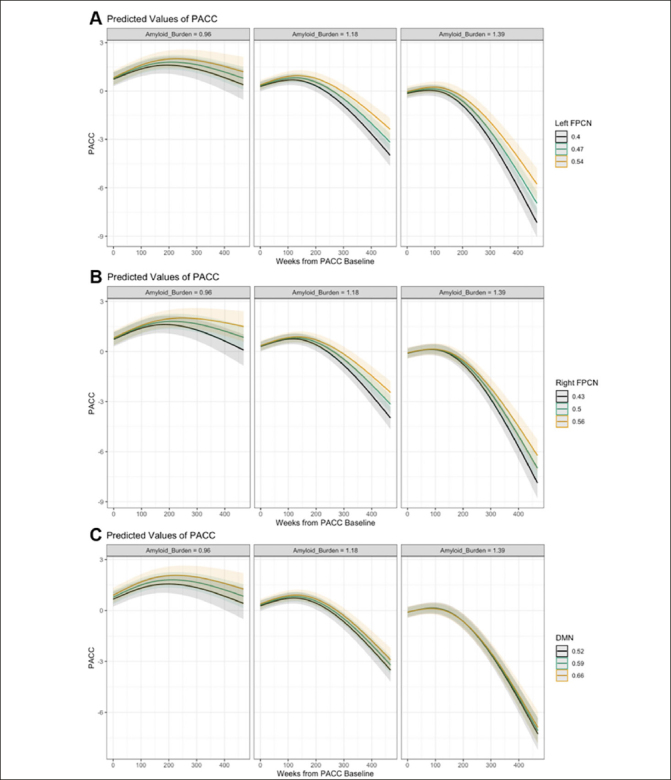
Marginal effects plots of the connectivity-by-time-by-Aβ interaction effect on PACC change (A: Left FPCN, B: Right FPCN, C: DMN) Note: Marginal effects of the connectivity-by-time-by-Aβ interaction are plotted here when cumulative dose is held constant at 0, continuous variables equal to their mean values and factor variables equal their reference values (ε4 status = non-carrier, group = A4 study; PACC version = Version A).

We observed a statistically significant three-way left FPCN-by-time-by-Aβ interaction effect on PACC change (p = .025) indicating a curvilinear relationship over time (see Supplementary Table 6 and 7 for likelihood ratio tests and full mixed effect models, respectively). That is, individuals with stronger functional connectivity of the left FPCN showed less cognitive decline over time at higher levels of Aβ compared with individuals who exhibited weaker connectivity (see Fig. 3A). These moderation effects were not significant for right FPCN (p = .709) and DMN (p = .643; see Figs. 3B-C). This pattern of results held across a series of sensitivity analyses (see Supplementary Results and Supplementary Table 8).

## Discussion

In a large multi-site clinical trial dataset in preclinical AD, stronger baseline functional connectivity of the left FPCN was associated with greater longitudinal cognitive resilience to baseline Aβ. In the entire A4 trial cohort, baseline Aβ levels were associated with greater cognitive decline (13) and this effect was also seen here in the placebo arm of the A4 trial and its natural history observational arm, the LEARN study. We observed that functional connectivity of the left FPCN moderated the negative effect of Aβ on longitudinal PACC change. This effect was such that individuals with stronger connectivity showed reduced Aβ-related cognitive decline.

Our finding extends previous cross-sectional findings where stronger connectivity, specific to the left FPCN, moderated the effect of AD pathology and AD-related neurodegeneration on cognitive performance (9–11). In this large cohort who are oversampled for preclinical AD and who are cognitively unimpaired at baseline, we show that this effect can be observed over a longer follow-up period, extending up to 9 years post-baseline. We also support our previous work suggesting that this effect is clearly observable in preclinical AD (8) whereas most previous studies included AD and MCI patients in their samples (9–11).

One major deviation from our previous study is that we had reported a protective effect for whole FPCN, as well as DMN, connectivity on Aβ-related cognitive decline(8). We replicated the FPCN effect but, in contrast to our earlier work, we did not observe a protective effect of DMN connectivity, even in relaxed models where we removed several important covariates. One possible explanation for this discrepancy is that the previously reported DMN effect may have reflected an effect of internetwork connectivity between the DMN and left FPCN, and therefore have been largely driven by connectivity of the left FPCN. Accordingly, previous work from our team has shown that the DMN-cognition association is largely mediated by FPCN connectivity (23). Other work has shown that connections between DMN and FPCN are associated with CR to neurodegeneration (5, 6, 24, 25). Therefore, while we previously observed a protective effect of DMN connectivity in earlier work, we offer the tentative explanation that this effect may have been driven by DMN-FPCN inter-network connectivity rather than a specific DMN effect and thus reflected the protective effect of the FPCN, which we observe in the present results.

Our finding of a CR effect specific to the FPCN supports previous findings (9) and also aligns with an emerging hypothesis, grounded in network control theory (26). Network control theory holds that particular hubs of the brain network are critical for enabling the brain to reach target states, as needed, in order to perform specific actions (27). Some target states may be easier to reach (analogous to performance of a cognitive task in the absence of network damage or dysfunction) and other states harder to reach (successful cognitive performance in the presence of network dysfunction due to pathology). Average control hubs help the brain to transition to multiple easy-to-reach states whereas modal control hubs enable transitions to difficult-to-reach states (26). Networks with high modal controllability may therefore be best able to support successful cognitive performance in the presence of brain dysfunction and pathology (26). In line with this idea, and our finding of a CR effect for the FPCN, but not DMN, the FPCN has been found to contain a high number of ‘modal control’ hubs whereas the DMN tends to contain ‘average control’ hubs (27). Future studies investigating associations between modal controllability in the FPCN and CR may help to provide more mechanistic insights into the nature of CR.

While we demonstrate that the protective effect of functional connectivity on Aβ-related cognitive decline is specific to the FPCN rather than the DMN, we also show that this effect is lateralized to the left FPCN. This finding confirms previous evidence of a left-lateralized effect (9). In a series of whole-brain analyses of task and resting-state data, the left lateral prefrontal cortex, a key hub of the left FPCN, was identified as a key functional hub that supports cognitive control via extensive global connectivity (28). The left FPCN has been shown to shift connectivity with task-relevant regions across the brain in response to different task demands and, in this way, supports adaptive cognitive performance (29). As such, when normal function of primary task-relevant regions or networks primary networks are impaired due to the accumulation of AD pathology, strong connectivity of the left FPCN may support successful cognitive performance by shifting connectivity to alternate or compensatory regions or networks.

While our analyses were well-powered, used an appropriate and flexible model for time, and were carefully adjusted for relevant confounds, there are some important caveats to our findings. First, the A4 and LEARN study participants who were eligible for Aβ-PET were highly educated, primarily white, and overall, in good health, with a truncated range of cognitive performance at baseline. Therefore this study cohort is not fully representative of the older population at risk for dementia in the US (15). Investigating the role of these networks in study cohorts that prioritize recruitment of under-represented groups, such as the AHEAD 3–45 Study (30) will therefore be necessary to assess the generalizability of these findings to more representative populations. Second, multi-site data is inevitable in clinical trials but introduces heterogeneity (i.e., scanner and acquisition) that can influence results and reliability (31). Future efforts could implement postdata acquisition methods to attenuate site and scanner effects, include site-wise de-meaning (31) or leave-site-out cross-validation (32). Here, we controlled for scanner using a random effect term in robust well-powered analyses that also adjusted for confounds including head motion. The regression coefficients for the spline terms in our statistical models are not directly interpretable and therefore we were restricted to graphical interpretation of the protective effect of left FPCN connectivity as compared to reporting an estimate of the effect size. Future work should attempt to clarify the magnitude of the protective effect of left FPCN connectivity on cognition. As CR to AD and co-pathologies, such as small vessel disease, is less prevalent than AD pathology alone (33), future work should also investigate the role of these networks in CR to AD and SVD pathology. Finally, it would be worthwhile to investigate whether similar effects are observed using functional outcomes (e.g. Instrumental Activities of Daily Living) to examine whether the left FPCN is also related to functional resilience (34).

In summary, in a large clinical trial cohort in preclinical AD, we observed a protective effect of left FPCN connectivity on longitudinal cognition where stronger connectivity was associated with reduced cognitive decline despite high levels of Aβ.

### Electronic Supplementary Material

Supplementary material, approximately 531 KB.

## Notes

*Ethical Standards:* Approval from an institutional review board was obtained at each of the sites and all participants and their study partners provided written informed consent prior to data collection.

## Notes

*Trial Registration Information:* ClinicalTrials.gov identifier: NCT02008357.
