# Supplementary material for: Left Frontoparietal Control Network Connectivity Moderates the Effect of Amyloid on Cognitive Decline in Preclinical Alzheimer's Disease: The A4 Study
Source: J Prev Alzheimers Dis. 2024 Jul 24;11(4):881–8. doi: 10.14283/jpad.2024.140 (PMC11266218; doi:10.14283/jpad.2024.140)
Supplement: Supplementary file 1 — Supplementary material, approximately 531 KB. [file mmc1.docx]

# 1. Supplementary Methods

## 1.1 Resting-state fMRI

Briefly, the first 4 volumes of each run were excluded prior to preprocessing. Each volume was realigned to the first volume of each run, directly normalized to MNI space using the SPM12 unified segmentation and normalization routine^1^, and spatially smoothed with a 6-mm FWHM Gaussian kernel. Participants were excluded from analysis if their scan met any of these poor quality assessment conditions: ≤ 50 volume-based signal-to-noise ratio; ≥ 0.5 mm mean framewise displacement (FWD); or DMN connectivity values < 0.3 (indicating a gross processing failure (e.g. highly inaccurate spatial normalization)).

## 1.2 Statistical analyses

The primary analysis in the A4 trial used natural cubic splines with 2 degrees of freedom, where the spline basis expansion assumes boundary knots at zero weeks and the maximum follow-up time and an interior knot at the median of observation times. This approach was applied in the A4 trial to accommodate the impact of COVID-19 on assessment timings and there are additional advantages to the use of splines in modeling cognitive change over time. Firstly, natural cubic splines enforce linearity before the first knot and after the last knot and therefore are less susceptible to overfitting at the extremes of the distribution^2,3^. Second, splines also allow for flexible modeling of temporal mean trends using fewer parameters than alternative models, such as mixed model repeated measures (MMRM) approach^4^. Finally, whereas each datapoint in a polynomial model affects the curve globally, the influence of each datapoint in a spline model is restricted to its respective spline, or segment^5^.

The following formulas summarize the two-way and three-way interaction effect models and the corresponding reduced models used in likelihood ratio tests:

- **Aβ-by-time model^:** PACC ~ (b_1_(t) + b_2_(t))×Aβ + BaselineAge + ε4Status + YearsEducation + AdjustedGMVolume + HeadMotion + StudyGroup + CumulativeDose + PACCVersion + (1 + t | Participant/Scanner)
- **Reduced Aβ model:** PACC ~ b_1_(t) + b_2_(t) + Aβ + BaselineAge + ε4Status + YearsEducation + AdjustedGMVolume + HeadMotion + StudyGroup + CumulativeDose + PACCVersion + (1 + t | Participant/Scanner)
- **FC-by-time model^***: PACC ~ (b_1_(t) + b_2_(t))×FC + Aβ + BaselineAge + ε4Status + YearsEducation + AdjustedGMVolume + HeadMotion + StudyGroup + CumulativeDose + PACCVersion + (1 + t | Participant/Scanner)
- **Reduced two-way FC model*:** PACC ~ (b_1_(t) + b_2_(t)) + FC + Aβ + BaselineAge + ε4Status + YearsEducation + AdjustedGMVolume + HeadMotion + StudyGroup + CumulativeDose + PACCVersion + (1 + t | Participant/Scanner)
- **FC-by-time-by-Aβ model^*:** PACC ~ FC×(b_1_(t) + b_2_(t))×Aβ + BaselineAge + ε4Status + YearsEducation + AdjustedGMVolume + HeadMotion + StudyGroup + CumulativeDose + PACCVersion + (1 + t | Participant/Scanner)
- **Reduced three-way FC model^*:** PACC ~ FC×(b_1_(t) + b_2_(t)) + ×(b_1_(t) + b_2_(t))×Aβ + BaselineAge + ε4Status + YearsEducation + AdjustedGMVolume + HeadMotion + StudyGroup + CumulativeDose + PACCVersion + (1 + t | Participant/Scanner)

*^ Full factorial model
* FC models were repeated separately for each network.*

where b_1_(t) and b_2_(t) are the spline basis expansion terms at observation time t.

Given their large units, adjusted GM volume and cumulative treatment dose variables were both scaled by dividing by 10,000. We conducted additional sensitivity analyses to fully interrogate the interaction effects in our models. In sensitivity analysis 1, we removed adjusted GM volume as a fixed effect in our primary model. In sensitivity analysis 2, we removed ε4 status, adjusted GM volume, and head motion as fixed effects from our primary model. In sensitivity analysis 3, we added the spline basis expansion of time-by-covariate interactions for all time-invariant covariates to our primary model.

# 2. Supplementary Results

**Supplementary Table 1. Maximum follow-up periods across participants.**

| **Maximum years of follow-up** | **N** |
| --- | --- |
| 0-0.99 | 52 |
| 1-1.99 | 62 |
| 2-2.99 | 60 |
| 3-3.99 | 70 |
| 4-4.99 | 76 |
| 5-5.99 | 175 |
| 6-6.99 | 291 |
| 7-7.99 | 206 |
| 8-9 | 29 |

**
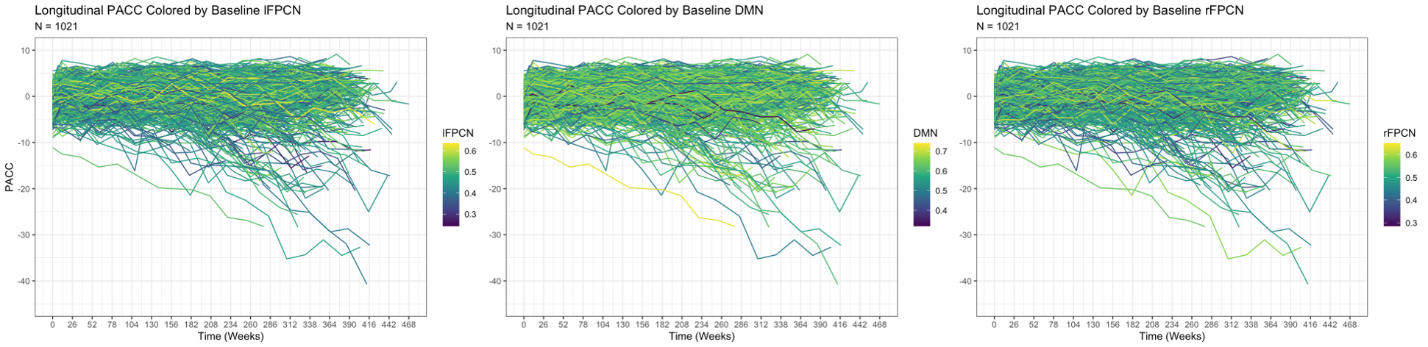
**

**Supplementary Figure 1.** Individual PACC trajectories colored by strength of functional connectivity in left FPCN, DMN, and right FPCN.

The three-way moderation effect was significant for the left FPCN only when we excluded adjusted GM volume as a covariate (sensitivity analysis 1, p = .024), when we restricted the covariates to baseline age, years of education, study group, and cumulative treatment dose (sensitivity analysis 2, p = .035), and when we included time-by-covariate interactions for all time-invariant covariates (sensitivity analysis 3, p = .027). See Supplementary Table 8 for likelihood ratio test results for these sensitivity analyses.

**Supplementary Table 2. Independent association of Aβ with PACC performance at baseline.**

| **Term** | **Estimate** | **CI** | **p-value** |
| --- | --- | --- | --- |
| (Intercept) | 9.516 | 7.27 – 11.76 | **<.001** |
| Aβ | -1.281 | -1.97 – -.59 | **<.001** |
| Age | -.144 | -.17 – -.11 | **<.001** |
| APOE ε4 status [Carrier] | -.023 | -.32 – .28 | .881 |
| Years of education | .084 | .04 – .13 | **<.001** |
| Head motion | -.749 | -2.63 – 1.13 | .434 |

| **FC** | **Term** | **Estimate** | **CI** | **p-value** |
| --- | --- | --- | --- | --- |
| Left FPCN | (Intercept) | 9.827 | 7.22 – 12.43 | **<.001** |
|  | Left FPCN | -.467 | -2.45 – 1.52 | .644 |
|  | Aβ | -1.287 | -1.98 – -.6 | **<.001** |
|  | Age | -.145 | -.17 – -.11 | **<.001** |
|  | APOE ε4 status [Carrier] | -.023 | -.32 – .28 | .878 |
|  | Years of education | .084 | .04 – .13 | **<.001** |
|  | Head motion | -.857 | -2.79 – 1.08 | .385 |
| Right FPCN | (Intercept) | 9.717 | 7.08 – 12.35 | **<.001** |
|  | Right FPCN | -.301 | -2.37 – 1.77 | .775 |
|  | Aβ | -1.282 | -1.97 – -.59 | **<.001** |
|  | Age | -.144 | -.17 – -.11 | **<.001** |
|  | APOE ε4 status [Carrier] | -.024 | -.32 – .27 | .873 |
|  | Years of education | .084 | .04 – .13 | **<.001** |
|  | Head motion | -.807 | -2.73 – 1.11 | .41 |
| DMN | (Intercept) | 9.253 | 6.48 – 12.03 | **<.001** |
|  | DMN | .32 | -1.67 – 2.31 | .752 |
|  | Aβ | -1.284 | -1.98 – -.59 | **<.001** |
|  | Age | -.143 | -.17 – -.11 | **<.001** |
|  | APOE ε4 status [Carrier] | -.021 | -.32 – .28 | .892 |
|  | Years of education | .085 | .04 – .13 | **<.001** |
|  | Head motion | -.689 | -2.60 – 1.23 | .48 |

**Supplementary Table 3. Independent associations of functional connectivity with PACC performance at baseline.**

**Supplementary Table 4. Aβ-by-time mixed effects model.**

| **Term** | **Estimate** | **CI** | **p-value** |
| --- | --- | --- | --- |
| (Intercept) | 12.703 | 8.325 – 17.082 | **<.001** |
| Aβ | -2.028 | -2.98 – -1.076 | **<.001** |
| b_1_(t) | 12.648 | 10.443 – 14.852 | **<.001** |
| b_2_(t) | 14.557 | 12.393 – 16.722 | **<.001** |
| Age | -.162 | -.192 – -.132 | **<.001** |
| APOE ε4 status [Carrier] | -.056 | -.335 – .223 | .695 |
| Years of education | .127 | .082 – .173 | **<.001** |
| Adjusted GM volume* | -.009 | -.057 – .039 | .717 |
| Head motion | .054 | -1.762 – 1.87 | .954 |
| Study group [LEARN] | -.244 | -.642 – .154 | .229 |
| Cumulative dose* | .681 | .256 – 1.106 | **.002** |
| PACC version B | -.219 | -.275 – -.164 | **<.001** |
| PACC version SC | -.976 | -1.043 – -.908 | **<.001** |
| Aβ:b_1_(t) | -11.424 | -13.281 – -9.566 | **<.001** |
| Aβ:b_2_(t) | -15.704 | -17.587 – -13.821 | **<.001** |

*Note: * Denotes scaled variables. Given their large units, adjusted GM volume and cumulative treatment dose variables were both scaled by dividing by 10,000. b_1_(t) and b_2_(t) are the spline basis expansion terms for time.*

**Supplementary Table 5. FC-by-time mixed effects models.**

| **Term** | **Left FPCN-by-time model** | | | **Right FPCN-by-time model** | | | **DMN-by-time model** | | |
| --- | --- | --- | --- | --- | --- | --- | --- | --- | --- |
|  | Estimate | CI | p | Estimate | CI | p | Estimate | CI | p |
| (Intercept) | 11.41 | 6.96 – 15.86 | **<.001** | 11.47 | 7.03 – 15.91 | **<.001** | 11.152 | 6.54 – 15.76 | **<.001** |
| FC | .852 | -1.05 – 2.76 | .38 | .55 | -1.44 – 2.54 | 0.59 | .882 | -1.01 – 2.78 | .362 |
| b_1_(t) | -5.131 | -7.99 – -2.27 | **<.001** | -4.62 | -7.8 – -1.44 | **.005** | -2.284 | -5.91 – 1.34 | .217 |
| b_2_(t) | -9.112 | -11.75 – -6.48 | **<.001** | -9.56 | -12.5 – -6.63 | **<.001** | -5.176 | -8.52 – -1.83 | **.002** |
| Aβ | -1.32 | -2.26 – -.38 | **.006** | -1.31 | -2.25 – -0.37 | **.006** | -1.33 | -2.27 – -.39 | **.005** |
| Age | -.161 | -.19 – -.13 | **<.001** | -.16 | -.19 – -0.13 | **<.001** | -.16 | -.19 – -.13 | **<.001** |
| APOE ε4 status [Carrier] | -.036 | -.32 – .24 | .8 | -.04 | -.32 – 0.24 | .791 | -.03 | -.31 – .25 | .835 |
| Years of education | .128 | .08 – .17 | **<.001** | .13 | .08 – 0.17 | **<.001** | .129 | .08 – .17 | **<.001** |
| Adjusted GM volume* | -.001 | -.06 – .04 | .713 | -.01 | -.06 – 0.04 | .774 | -.009 | -.06 – .04 | .727 |
| Head motion | .076 | -1.78 – 1.93 | .936 | -.03 | -1.87 – 1.81 | .973 | .153 | -1.69 – 2 | .871 |
| Study group [LEARN] | -.17 | -.57 – .23 | .402 | -.16 | -.57 – 0.23 | .419 | -.166 | -.56 – .23 | .414 |
| Cumulative dose* | -.764 | -1.13 – -.4 | **<.001** | -.78 | -1.14 – -0.41 | **<.001** | -.763 | -1.13 – -.4 | **<.001** |
| PACC version B | -.213 | -.27 – -.16 | **<.001** | -.21 | -.27 – -0.16 | **<.001** | -.213 | -0.27 – -.16 | **<.001** |
| PACC version SC | -.988 | -1.1 – -.92 | **<.001** | -.99 | -1.1 – -0.92 | **<.001** | -.988 | -1.06 – -.92 | **<.001** |
| FC:b_1_(t) | 9.753 | 3.73 – 15.77 | **.002** | 8.2 | 1.85 – 14.54 | **.011** | 2.949 | -3.16 – 9.06 | .344 |
| FC:b_2_(t) | 12.762 | 7.23 – 18.3 | **<.001** | 12.98 | 7.14 – 18.83 | **<.001** | 3.5 | -2.14 – 9.14 | .224 |

*Note: * Denotes scaled variables. Given their large units, adjusted GM volume and cumulative treatment dose variables were both scaled by dividing by 10,000. b_1_(t) and b_2_(t) are the spline basis expansion terms for time.*

**Supplementary Table 6. Likelihood ratio tests of mixed effects models showing the Aβ-by-time, FC-by-time, and FC-by-time-by-Aβ interaction effects on PACC change over time.**

| **Model** | **AIC** | **Log-likelihood** | **LRT vs Reduced model** | | |
| --- | --- | --- | --- | --- | --- |
|  |  |  | **Test statistic** | **df** | **p-value** |
| Aβ-by-time | 48,689.1 | -24,324.6 | 257.5 | 22 | <.001 |
| Reduced | 48,435.6 | -24,195.8 | - | 20 | - |
| Left FPCN-by-time | 48,673.2 | -24,313.6 | 21.8 | 23 | <.001 |
| Reduced | 48,691 | -24,324.5 | - | 21 | - |
| Left FPCN-by-time-by-Aβ | 48,423.4 | -24,183.7 | 7.4 | 28 | .025 |
| Reduced | 48,426.8 | -24,187.4 | - | 26 | - |
| Right FPCN-by-time | 48,671.6 | -24,324.5 | 23.5 | 23 | <.001 |
| Reduced | 48,691.1 | -24,312.8 | - | 21 | - |
| Right FPCN-by-time-by-Aβ | 48,426.4 | -24,185.2 | .7 | 28 | .709 |
| Reduced | 48,423.1 | -24,185.6 | - | 26 | - |
| DMN-by-time | 48,690.5 | -24,324.2 | 1.5 | 23 | .472 |
| Reduced | 48,692 | -24,323.5 | - | 21 | - |
| DMN-by-time-by-Aβ | 48,440.3 | -24,194.1 | .9 | 28 | .643 |
| Reduced | 48,443.4 | -24,193.7 | - | 26 | - |

*AIC: Akaike Information Criterion, LRT: Log-likelihood ratio test, df: degrees of freedom.*

**Supplementary Table 7. FC-by-time-by-Aβ mixed effects models.**

| **Term** | **Left FPCN-by-time-by-Aβ model** | | | **Right FPCN-by-time-by-Aβ model** | | | **DMN-by-time-by-Aβ model** | | |
| --- | --- | --- | --- | --- | --- | --- | --- | --- | --- |
|  | Estimate | CI | p | Estimate | CI | p | Estimate | CI | p |
| (Intercept) | 12.459 | 5.91 – 19 | **<.001** | 11.659 | 4.71 – 18.6 | **.001** | 9.495 | 1.93 – 17.06 | .014 |
| FC | .356 | -9.84 – 10.55 | .945 | 1.859 | -8.91 – 12.63 | .735 | 5.177 | -5.15 – 15.5 | .326 |
| b_1_(t) | 15.609 | .89 – 30.33 | **.038** | 9.33 | -7.17 – 25.83 | .268 | 5.141 | -14 – 24.29 | .599 |
| b_2_(t) | 24.405 | 10.7 – 38.11 | **<.001** | 12.524 | -3.01 – 28.06 | .114 | 11.542 | -6.46 – 29.54 | .209 |
| Aβ | -2.174 | -6.19 – 1.84 | .289 | -1.427 | -5.94 – 3.09 | .535 | .107 | -5.01 – 5.23 | .967 |
| Age | -.161 | -.19 – -.13 | **<.001** | -.162 | -.19 – -0.13 | **<.001** | -.16 | -.19 – -.13 | <.001 |
| APOE ε4 status [Carrier] | -.056 | -.34 – .22 | .693 | -.061 | -.34 – 0.22 | .669 | -.052 | -.33 – .23 | .715 |
| Years of education | .128 | .08 – .17 | **<.001** | .127 | .08 – 0.17 | **<.001** | .127 | .08 – .17 | <.001 |
| Adjusted GM volume* | -.009 | -.06 – .04 | .703 | -.007 | -.06 – 0.04 | .765 | -.009 | -.06 – .04 | .718 |
| Head motion | .126 | -1.73 – 1.98 | .894 | .026 | -1.82 – 1.87 | .978 | .223 | -1.62 – 2.07 | .813 |
| Study group [LEARN] | -.25 | -.65 – .15 | .219 | -.244 | -.64 – 0.15 | .23 | -.245 | -.64 – .15 | .228 |
| Cumulative dose* | .648 | .22 – 1.07 | **.003** | .648 | .22 – 1.07 | **.003** | .673 | .25 – 1.1 | .002 |
| PACC version B | -.219 | -.27 – -.16 | **<.001** | -.219 | -.28 – -0.16 | **<.001** | -.219 | -.27 – -.16 | <.001 |
| PACC version SC | -.976 | -1.04 – -.91 | **<.001** | -.976 | -1.04 – -0.91 | **<.001** | -.976 | -1.04 – -.91 | <.001 |
| FC:b_1_(t) | -6.818 | -37.98 – 24.34 | .668 | 6.496 | -26.48 – 39.48 | .7 | 12.755 | -19.6 – 45.11 | .44 |
| FC:b_2_(t) | -21.71 | -50.7 – 7.27 | .142 | 3.807 | -27.21 – 34.82 | .81 | 5.13 | -25.26 – 35.52 | .741 |
| FC:Aβ | .318 | -8.16 – 8.8 | .941 | -1.194 | -10.17 – 7.78 | .794 | -3.656 | -12.23 – 4.9 | .403 |
| b_1_(t):Aβ | -17.013 | -29.28 – -4.74 | **.007** | -11.438 | -25.22 – 2.35 | .104 | -6.332 | -22.31 – 9.65 | .438 |
| b_2_(t):Aβ | -28.116 | -39.49 – -16.74 | **<.001** | -18.715 | -31.64 – -5.78 | **.005** | -14.802 | -29.81 – .21 | .054 |
| FC:b_1_(t):Aβ | 12.356 | -13.71 – 38.42 | .353 | .192 | -27.42 – 27.81 | .989 | -8.657 | -35.68 – 18.36 | .53 |
| FC:b_2_(t):Aβ | 27.113 | 2.99 – 51.24 | **.028** | 6.323 | -19.52 – 32.16 | .632 | -1.542 | -26.87 – 23.79 | .91 |

*Note: * Denotes scaled variables. Given their large units, adjusted GM volume and cumulative treatment dose variables were both scaled by dividing by 10,000. b_1_(t) and b_2_(t) are the spline basis expansion terms for time.*

**Supplementary Table 8. Likelihood ratio tests for sensitivity analyses of three-way FC-by-time-by-Aβ interaction effects.**

| **Sensitivity Analysis** | **Model** | **AIC** | **Log-likelihood** | **LRT vs Reduced model** | | |
| --- | --- | --- | --- | --- | --- | --- |
|  |  |  |  | **Test statistic** | **Df** | **p-value** |
| #1: Adjusted GM vol. removed as a covariate | Left FPCN-by-time-by-Aβ | 48,421.5 | -24,183.8 | 7.4 | 27 | .024 |
|  | Reduced | 48,425 | -24,187.5 | - | 25 | - |
|  | Right FPCN-by-time-by-Aβ | 48,424.5 | -24,185.26 | .7 | 27 | .708 |
|  | Reduced | 48,421.2 | -24,185.6 | - | 25 | - |
|  | DMN-by-time-by-Aβ | 48,441.5 | -24,193.8 | .883 | 27 | .643 |
|  | Reduced | 48,438.4 | -24,194.2 | - | 25 | - |
| #2: Covariates restricted to age, education, study group, treatment dose | Left FPCN-by-time-by-Aβ | 49,188.9 | -24,571.4 | 6.7 | 23 | .035 |
|  | Reduced | 49,191.6 | -24,574.8 | - | 21 | - |
|  | Right FPCN-by-time-by-Aβ | 49,191.7 | -24,572.83 | .5 | 23 | .77 |
|  | Reduced | 49,188.2 | -24,573.1 | - | 21 | - |
|  | DMN-by-time-by-Aβ | 49,207.8 | -24,580.9 | .9 | 23 | .6 |
|  | Reduced | 49,204.7 | -24,581.4 | - | 21 | - |
| #3: All time-by-covariate interactions included for time-invariant covariates | Left FPCN-by-time-by-Aβ | 48,357.1 | -24,138.5 | 7.2 | 40 | .027 |
|  | Reduced | 48,360.3 | -24,142.1 | - | 38 | - |
|  | Right FPCN-by-time-by-Aβ | 48,363.3 | -24,141.6 | .3 | 40 | .88 |
|  | Reduced | 48,359.5 | -24,141.8 | - | 38 | - |
|  | DMN-by-time-by-Aβ | 48,372.6 | -24,146.3 | .9 | 40 | .642 |
|  | Reduced | 38,369.5 | -24,146.7 | - | 38 | - |

*Note: AIC: Akaike Information Criterion, LRT: Log-likelihood ratio test, df: degrees of freedom. b_1_(t) and b_2_(t) are the spline basis expansion terms for time.*

# 3. Supplementary References

1. Calhoun VD, Wager TD, Krishnan A, et al. The impact of T1 versus EPI spatial normalization templates for fMRI data analyses. *Human Brain Mapping*. 2017;38(11):5331-5342. doi:10.1002/HBM.23737

2. Perperoglou A, Sauerbrei W, Abrahamowicz M, Schmid M. A review of spline function procedures in R. *BMC Medical Research Methodology*. 2019;19(1):46. doi:10.1186/s12874-019-0666-3

3. Elhakeem A, Hughes RA, Tilling K, et al. Using linear and natural cubic splines, SITAR, and latent trajectory models to characterise nonlinear longitudinal growth trajectories in cohort studies. *BMC Medical Research Methodology*. 2022;22(1):68. doi:10.1186/s12874-022-01542-8

4. Donohue MC, Langford O, Insel PS, et al. Natural cubic splines for the analysis of Alzheimer’s clinical trials. *Pharmaceutical Statistics*. 2023;22(3):508-519. doi:10.1002/pst.2285

5. Faraway JJ. *Linear Models with R*. 2nd ed. Chapman and Hall/CRC; 2016. doi:10.1201/b17144
